# Fabrication of Microfiber Patterns with Ivy Shoot-Like Geometries Using Improved Electrospinning

**DOI:** 10.3390/ma9040266

**Published:** 2016-04-01

**Authors:** Young Hun Jeong, Jongwan Lee

**Affiliations:** 1 School of Mechanical Engineering, Kyungpook National University, 80 Daehakro, Bukgu, Daegu 41566, Korea; 2 Department of Mechanical Engineering, Ulsan National Institute of Science and Technology (UNIST), 50 UNIST-gil, Eonyang-eup, Ulsan 44919, Korea; jwlee@unist.ac.kr

**Keywords:** electrospinning, microfibers, nanofibers, direct-writing, ivy shoot-like pattern, fractals

## Abstract

Fibers and fibrous structures are used extensively in various fields due to their many advantages. Microfibers, as well as nanofibers, are considered to be some of the most valuable forms of advanced materials. Accordingly, various methods for fabricating microfibers have been developed. Electrospinning is a useful fabrication method for continuous polymeric nano- and microfibers with attractive merits. However, this technique has limitations in its ability to control the geometry of fibrous structures. Herein, advanced electrospinning with direct-writing functionality was used to fabricate microfiber patterns with ivy shoot-like geometries after experimentally investigating the effects of the process conditions on the fiber formation. The surface properties of the fibers were also modified by introducing nanoscale pores through the use of higher levels of humidity during the fabrication process.

## 1. Introduction

Various fibers and fibrous structures are attracting significant attention in many different fields, such as cosmetics, clothing, electronics, environmental, chemical, and nano- and bioengineering [1]. Well-known nanofibers are a promising advanced material, but microfibers, a fiber form having a different order of scale, are also used widely in various areas, such as tissue engineering [2,3,4], drug delivery [5,6], filtration [7,8], and composite reinforcement [9], owing to their numerous advantages, including high porosity, large pore size, large surface area-to-volume ratio, high flexibility, and similarity in structural form with the human body’s extracellular matrices. In particular, microfibers have yielded better quality results when compared to nanofibers; e.g., microfiber and multilayered scaffolds have improved quality and perform better in terms of initial cell attachments and cell infiltration processes compared with nanofibrous scaffolds in tissue engineering [3].

To date, various methods have been developed to fabricate microfibers. In general, spinning methods such as wet-, dry-, and gel-spinning processes have been used successfully to fabricate microfibers for various applications [10,11,12,13,14]. Various types of microfluidic chips have also been used as nozzles for extrusion with versatile functionalization, such as Janus structures with multiple materials and bubbling [2,4,15,16]. In particular, the electrohydrodynamic hot-jet plotting process has been introduced to microfiber fabrication, providing better controllability and fiber diameters as small as 5 μm [17]. A slit die extrusion and heat stretching process for fabricating microfibers has also been demonstrated [18].

Electrospinning is one of the most attractive methods for fabricating continuous polymeric fibers with diameters ranging from micro- to nanometer scales because this technique does not pose serious limitations on the material selection and requires simple fabrication equipment with relatively easy process operations [1]. Therefore, various fibers and fibrous structures with different sizes, geometries, and materials have been fabricated using electrospinning and applied to a variety of fields. Especially, Pham *et al.* reported that the diameter of electrospun microfibers could be accurately controlled through the proper selection of process parameters, such as electric field, concentration, and flow rate [3]. Fridrikh *et al.* identified the relationship between fiber diameter, surface tension, flow rate, and electric current in the jet [19]. McKee *et al.* investigated the effects of concentration/viscosity on electrospun fiber diameter [20]. However, the conventional electrospinning process has limitations in terms of the geometric control of fibrous structures owing to the whipping motion that results from the bending instability of the electrospinning jet [21]. In this regard, conventional electrospinning is only suitable for fabricating randomly deposited nonwoven fibrous meshes, even though uniaxially aligned fibrous mats can be fabricated using electrospinning with a drum collector [22]. Many approaches have been tried to address this problem and obtain fibrous structures having regular shapes [23,24,25,26,27,28].

In the current study, microfibrous patterns with ivy shoot-like geometries were fabricated using an improved electrospinning process, known as direct-write electrospinning (DWES), which was developed in our previous study [29]. Since DWES improves the geometric regularity of electrospun fibers in a controllable manner, it was used to fabricate electropsun microfiber patterns to overcome their geometric control limitation. The effects of the experimental conditions, such as flow rate, scan speed, and humidity, on the microfiber formation were investigated using Euclidean and fractal analyses. As a result, we demonstrated that controllable microfiber patterns could be fabricated using the proposed electrospinning process, yielding complex and random ivy shoot-like shapes in the microfiber patterns. In addition, the surface properties could be modified by using higher levels of humidity in the fabrication process.

## 2. Results and Discussion

### 2.1. Influence of the Solution Flow Rate on the Microfibers

Polymeric fibers were electrospun under various solution flow rate conditions because this parameter was considered to be one of the dominant factors that influence the electrospun microfiber diameter [30]. In the experiments, the fabricated pattern had a lattice shape with a grid size of 500 μm, and the flow rates of the polycaprolactone (PCL) solution from the nozzle were 0.1, 0.3, 0.6 and 0.8 mL/h. Other experimental conditions were kept nearly constant, as indicated in Table 1. The tip-to-collector distance (TCD) and voltage for electrospinning were 60.0 mm and 21–24 kV, respectively.

**Figure 1:**
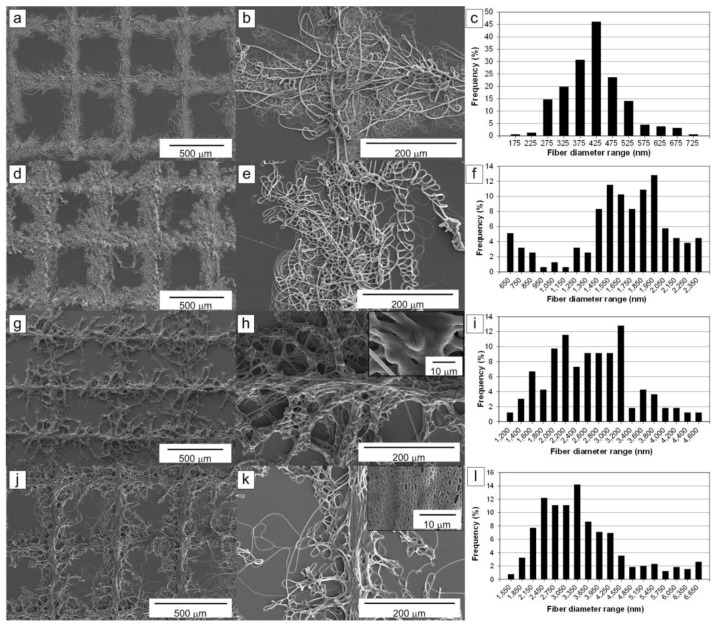
Scanning electron microscopy (SEM) images and fiber diameter distributions of the nano- and microfibrous lattice patterns fabricated with various solution flow rates: (**a**–**c**) 0.1 mL/h; (**d**–**f**) 0.3 mL/h; (**g**–**i**) 0.6 mL/h; and (**j**–**l**) 0.8 mL/h.

Figure 1 shows the fibrous lattice patterns fabricated under various flow rates, and their fiber diameter distributions. The fibrous pattern fabricated with a flow rate of 0.1 mL/h is depicted in Figure 1a,b. It contained many fibers with diameters ranging from 150 nm to 5 μm. However, although plenty of microfibers are shown in Figure 1b, the nanometer-scale fibers still dominated the pattern. A well-defined lattice pattern was obtained under these conditions. The most common fiber diameter was 425 nm, as depicted in Figure 1c.

Figure 1d–f show the pattern and fiber diameter distribution obtained using a flow rate of 0.3 mL/h. The fibrous line pattern width of the lattice pattern increased because of the higher flow rate, and most of the fibers had microscale diameters. The most common fiber diameter was 2 μm, and the minimum diameter was 600 nm. The pattern still exhibited a regular lattice shape. Thus, the higher flow rate provided larger-diameter fibers.

The fiber diameter continued to increase with the flow rate. A unique morphology of the fibrous pattern was observed at a flow rate greater than 0.6 mL/h. Figure 1g,h show the lattice pattern obtained at a flow rate of 0.6 mL/h. In Figure 1g, the pattern had a unique geometry that looked like ivy shoots on a wall. The stems of the ivy shoot-like pattern (central lines) still maintained the lattice shape. However, tendril-like microfibers stretched radially from the stem lines and caused the pattern to resemble ivy shoots. The fibers had larger diameters and were fused together to create the complex morphology shown in Figure 1h, likely because a significant amount of the solvent remained in the electrospun fibers after they reached the collector plate. Therefore, the pattern had better interconnectivity among fibers compared with Figure 1b,e. Also, the width of the line pattern became narrower even though the solution flow rate was higher, and a few pores were exposed on the surface of the fibers due to incomplete evaporation of solvent. As shown in Figure 1i, the most common fiber diameter was between 2.2 and 3.2 μm.

The fiber interconnectivity continued to improve when the solution flow rate was increased to 0.8 mL/h due to a higher degree of fiber fusion, as shown in Figure 1j,k. However, the ivy shoot-like geometric features became weaker, as depicted in Figure 1k. As expected, the porosity of the fiber surfaces increased significantly. From Figure 1l, the most common microfiber diameter was 3.35 μm.

From the results shown in Figure 1, the fabricated nano- and microfibrous patterns had well-defined lattice shapes with various line patterns, depending on the solution flow rate used. Especially, it could be seen that the clearest ivy shoot pattern could be obtained at a flow rate of 0.6 mL/h, as shown in Figure 1g. However, at flow rates greater than 0.9 mL/h, it was difficult to obtain the ivy shoot pattern from the electrospun fibers.

**Figure 2:**
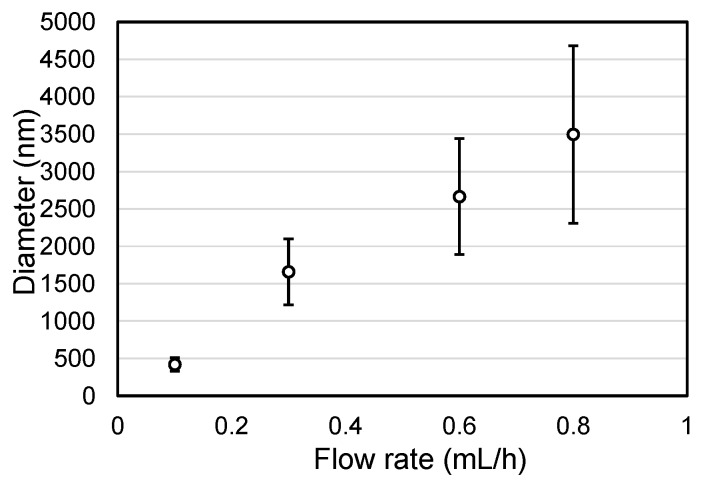
Relationship between solution flow rate and fiber diameter.

The relationship between the solution flow rate and the fiber diameter of the fabricated lattice patterns shown in Figure 1 was obtained via statistical analysis. As illustrated in Figure 2, the average fiber diameter of the fabricated patterns increased with the solution flow rate. Thus, the average diameter of the electrospun fibers could be controlled by modifying the solution flow rate. However, the higher flow rates resulted in larger standard deviations among fiber diameters.

### 2.2. Influence of the Scan Speed of the Collector

**Figure 3:**
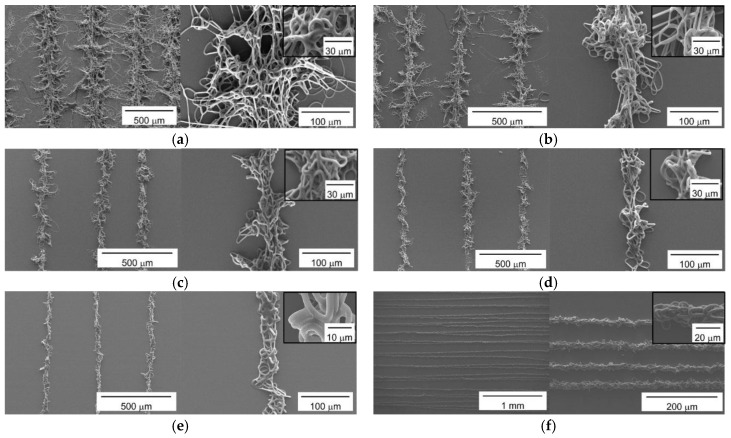
Influence of the scan speed of the collector on the shape morphology of the fractal geometric microfibrous patterns: SEM images of the fabricated microfibrous patterns at scan speeds of (**a**) 10; (**b**) 25; (**c**) 50; (**d**) 75; (**e**) 150; and (**f**) 450 mm/s. The scan path distance of fabricated microfibrous patterns was (**a**–**e**) 500 μm and (**f**) 50 μm.

In the experiments described in the previous section, we observed that the lattice patterns consistently possessed ivy shoot-like geometries when the solution flow rate was between 0.6 and 0.8 mL/h. Next, we modified the scan speed of the collector to adjust the degree of complexity of the ivy shoot-like geometries in the microfibrous patterns, because extending the length of time that the collector remained at one point could increase the possibility for a tendril to form. Thus, parallel-line patterns were fabricated using various collector scan speeds from 10 to 450 mm/s, as shown in Figure 3. The other conditions were fixed as listed in Table 2. The scan path used to fabricate the parallel-line pattern had a pitch of 500 μm at all scan speeds except for 450 mm/s, for which the pitch was 50 μm.

Figure 3a shows the parallel-line pattern fabricated at a scan speed of 10 mm/s. The central lines corresponding to the stem in an ivy shoot followed the scan path well with a regular pitch. Moreover, the pattern clearly showed complex tendril formation. The fabricated microfibrous patterns obtained at scan speeds of 20, 50 and 75 mm/s are presented in Figure 3b–d, respectively. The formation of tendrils in the microfibrous line patterns decreased gradually as the scan speed increased. At the same time, the width of the line patterns narrowed. In Figure 3e, which shows the pattern fabricated at a scan speed of 150 mm/s, it is difficult to identify any clear tendrils along the line patterns. Moreover, we obtained a straight-line pattern without tendrils at a scan speed of 450 mm/s, as shown in Figure 3f, with highly arranged entanglements. Thus, the degree of complexity obtained using the proposed electrospinning process could be controlled via changes in the scan speed. The inferred explanation for this result is that the longer durations spent by the collector at a given point due to the slower scan speeds introduced a greater possibility for the microfibrous pattern to extend tendrils (fingers).

**Figure 4:**
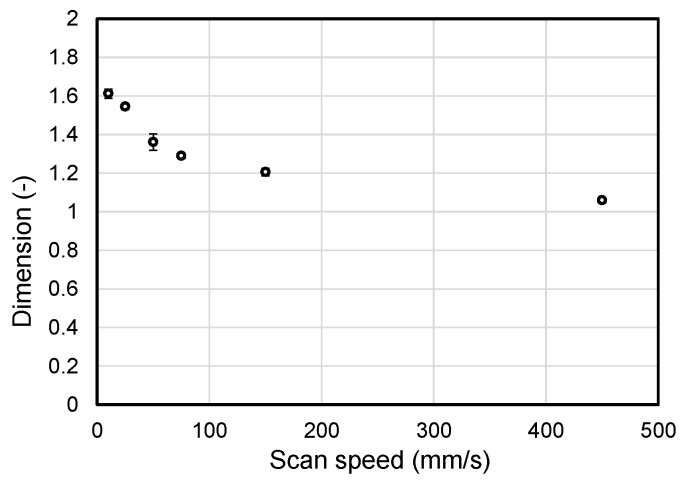
Influence of the scan speed of the collector on the fractal dimension of the ivy shoot-like microfibrous patterns.

The effect of the scan speed on the tendril formation in the fabricated microfibrous patterns was evaluated by the well-defined fractal dimension, which could describe complexity or area filling of chaotic geometries in nature [31,32]. We calculated fractal dimensions of the patterns for various scan speed conditions. Figure 4 shows fractal dimension of line patterns fabricated under the conditions mentioned in Table 2 and Figure 3. The fractal dimension of the line pattern fabricated at a scan speed of 10 mm/s was about 1.612 with a standard deviation of about 0.024. The dimension decreased as the scan speed increased, thus it became down to 1.205 ± 0.019 when the scan speed was 150 mm/s. Especially, the fractal dimension of the pattern fabricated at a scan speed of 450 mm/s was 1.059 ± 0.011, which was close to 1.0, corresponding to the typical one-dimensional geometry (*i.e*., simple line). In this regard, it could be concluded from the figure that the fractal dimension of the microfibrous patterns at flow rate of 0.6 mL/h ranged between 2.0 and 1.0 and the slower scan speed led the higher fractal dimension.

**Figure 5:**
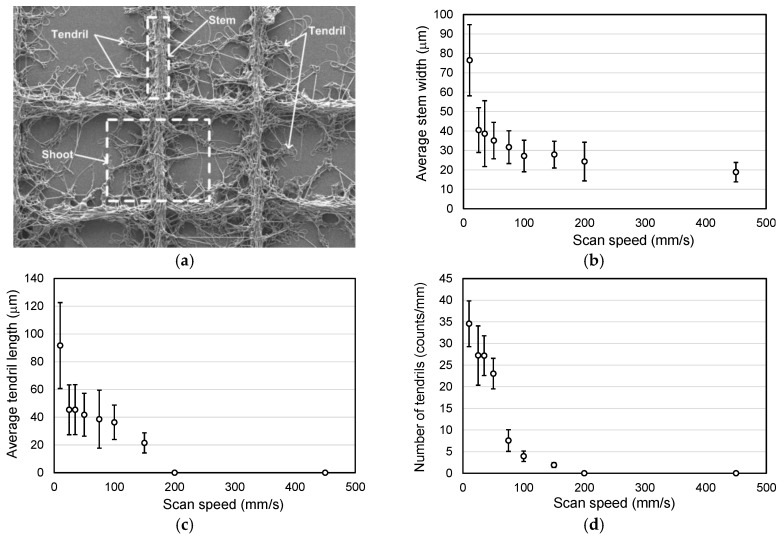
(**a**) Explanatory image showing how the fabricated fractal geometric microfibrous patterns consist of stems, tendrils, and shoots. Relationships between the scan speed of the collector and the (**b**) average stem width; (**c**) average tendril length; and (**d**) number of tendrils.

The characteristics of the ivy shoot-like geometries in the fabricated microfibrous patterns were analyzed to more clearly identify the effect of scan speed, as shown in Figure 5. First, we established a simple measure of the degree of complexity of a fractal geometry in the microfibrous line patterns. The shapes in Figure 5a, which presents a typical fractal geometry in a fabricated microfibrous pattern, resemble ivy shoots on a wall, and thus the geometric characteristics were defined according to the geometry of ivy shoots. The line patterns of microfibers with fractal geometries were therefore identified as shoots in this study. The main line patterns corresponding to the scan path were identified as stems, and the branches from the stems were identified as tendrils. Therefore, a shoot consisted of a stem and many tendrils. Figure 5b illustrates the relationship between the stem width and the scan speed. The width narrowed with increasing scan speed. The line width ranged between 20 and 30 μm at scan speeds greater than 100 mm/s; in particular, the width could be reduced to 20 μm when the scan speed was 450 mm/s. Also, the line widths tended to have larger standard deviations at lower scan speeds because the instability of the deposition increased. The effects of the scan speed on the length and number of tendrils showed trends similar to the stem width, as shown in Figure 5c,d. The tendrils extended to about 90 μm on average; however, no tendrils were identified in the samples fabricated with scan speeds greater than 200 mm/s. Consequently, the complexity of the fractal geometries in a fabricated microfibrous mat, which could be characterized by the average width of the stems, and the number and average length of the tendrils, could be controlled via the scan speed.

### 2.3. Influence of the Relative Humidity

Porosity can be introduced to the surface of electrospun micro- or nanofibers by controlling the humidity. This is because the humidity can influence the evaporation of solvent from electrospun fibers. Nanopores introduced to microfibers can affect various surface properties. Thus, parallel-line patterns with a line pitch of 500 μm were fabricated at two different levels of relative humidity, 51% and 60%, which was selected from preliminary tests and the experiments mentioned at Table 1 and Table 2. The higher humidity led to more pores on the microfiber surface under the flow rate fixed at 0.6 mL/h. However, clear pores could not be observed at humidity lower than 60 percent relative humidity (RH%). When humidity was 60 RH%, clear pores covering the surface could be obtained. However, the amount of electrospun fibers significantly decreased when the humidity was higher than 60 RH%. The experimental conditions are listed in Table 3.

**Figure 6:**
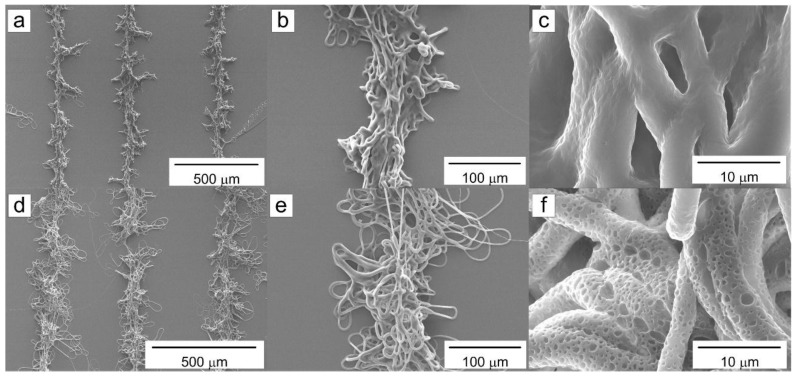
SEM images of fractal geometric microfibrous patterns obtained at different levels of relative humidity: (**a**–**c**) 51%, and (**d**–**f**) 60%.

**Figure 7:**
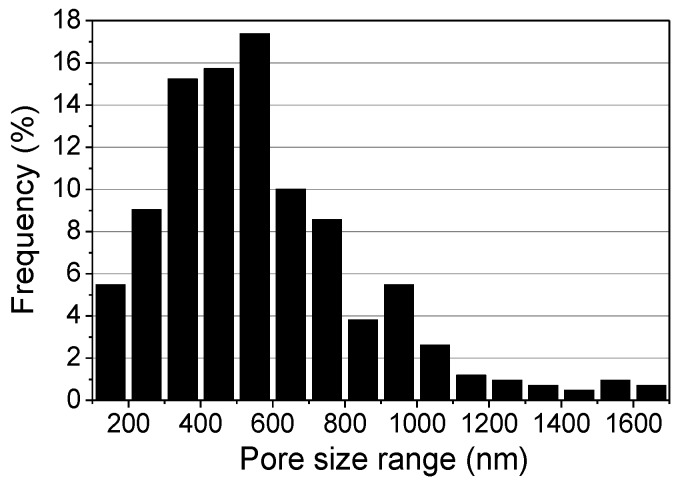
Pore size distribution of the fractal geometric microfibrous pattern shown in Figure 6d–f obtained at 60% relative humidity.

All patterns exhibited fractal geometries, regardless of the relative humidity. However, the pattern fabricated at the lower humidity that had no pores on its surface (Figure 6a–c), unlike the pattern fabricated at higher humidity was covered by micro- and nanopores (Figure 6d–f). The diameter distribution of the pores covering the surface of the pattern shown in Figure 6f is given in Figure 7. Most of the pores had diameters ranging between 200 and 800 nm, and the most common pore diameter was about 550 nm. The average pore diameter was 599.3 nm.

## 3. Experimental Section

**Figure 8:**
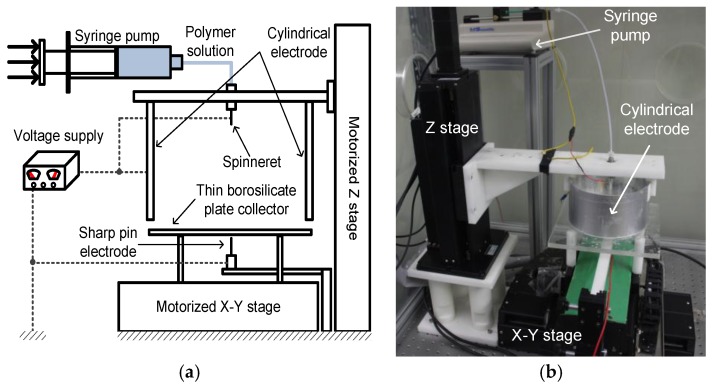
Electrospinning setup for fabricating microfiber patterns: (**a**) Schematic diagram of the direct-write electrospinning (DWES) apparatus and (**b**) actual DWES apparatus.

The fabrication of microfiber patterns with regular geometries requires an improved electrospinning apparatus that can focus the electrospinning jet and provide a scanning motion for fiber deposition. Thus, we used a DWES experimental setup, which was developed in our previous study [29]. Figure 8a shows a schematic diagram of the DWES setup, which was based on a conventional electrospinning apparatus composed of a syringe pump, high-voltage supply, micronozzle, and conductive collector plate. However, in this study, the conductive collector plate in the conventional electrospinning setup was replaced with a sharp-pin grounded electrode and a thin borosilicate plate collector with a thickness of about 150 μm. Also, an aluminum-coated cylindrical electrode was introduced to enhance the performance of the electrospinning jet focusing. The borosilicate plate was given a planar motion with the help of a motorized XY stage equipped with linear servo motors, while the sharp-pin grounded electrode was fixed to ground. The tip-to-collector distance (TCD) was controlled using a vertical motorized stage, while the distance between the collector and the sharp pin was fixed at about 20 μm using a manual stage. Figure 8b shows the actual electrospinning setup used in this study. The electrospinning apparatus was isolated using an acrylic chamber, and thus the atmospheric conditions, such as temperature and relative humidity, could be controlled at consistent levels. The polymer used to fabricate the microfibers was a polycaprolactone with an average molecular weight ranging between 70,000 and 90,000 (PCL, 440744, Sigma-Aldrich, Co., St. Louis, MO, USA), which was dissolved in 99.5% pure chloroform (C0584, Samchun Pure Chemical Co., Ltd., Seoul, Korea) at a concentration of 8.8 wt % via stirring for 120 min.

SEM images shown in Figure 1, Figure 3, Figure 5 and Figure 6 were acquired using a field-emission scanning electron microscope (S4700, Hitachi Co., Tokyo, Japan), after the fabricated samples were sputter-coated with platinum (E-1030, Hitachi Co.). The fiber diameter analyses were performed using ImageJ program (NIH). For the analysis, we used three different samples and an exemplary SEM image with a size of 120 μm × 160 μm was selected for each sample. The number of measurements of fiber diameter for each image ranged between 155 and 648, which depended on fiber size and pattern quality. The fractal dimensions were analyzed using the Fractalyse program [33]. In the calculation of fractal dimension of the pattern at each condition, three different line pattern samples were used. Especially, the SEM image for each sample was converted to black (fibers) and white (background) image using image processing for the Fractalyse program.

## 4. Conclusions

We demonstrated that microfibrous patterns with fractal geometries that looked like ivy shoots on a wall could be successfully fabricated using an advanced electrospinning method with specific process conditions. A direct-write electrospinning method consisting of an additional cylindrical side-wall electrode, a dielectric thin collector plate with planar motion, and a sharp-pin grounded electrode, as well as a conventional electrospinning apparatus, was employed in the fabrication to introduce focusing and scanning functionalities to the electrospinning jet. As a result, fibrous patterns with regular shapes were successfully fabricated. The solution flow rate, scan speed of the collector, and relative humidity were considered as key parameters in the fabrication of the microfibrous patterns with fractal geometries. The solution flow rate directly influenced the fiber diameter; microscale fibers were obtained using flow rates greater than 0.3 mL/h. The fractal geometries were introduced to the fibrous patterns by using collector scan speeds that were less than 100 mm/s with a flow rate that was greater than 0.6 mL/h. The scan speed strongly influenced the dimensions and complexity of the fractal geometries. Higher levels of humidity introduced nanoscale pores on the surface of the microfibers, which could offer a variety of functionalities.

The ivy shoot-like pattern presented in this study can be used in various applications related to tissue engineering, biochip application, and drug delivery. Its more-natural geometry can give cells in culture a positive effect on guidance toward more realistic engineered tissues. It can be used as a mold master for microfluidic biochip which can introduce more realistic vascular network or surface properties. Also, it would be a good drug carrier because of its area filling property, which is a well-known feature of fractal geometry.

## Figures and Tables

**Table 1:** Experimental conditions for fabricating a fibrous lattice pattern under various flow rate conditions.

Parameter (Units)	Value
Polymer-solvent (concentration, wt %)	PCL-chloroform (8.8 wt %)
Temperature (°C)	21–22
Relative humidity (%)	55–58
Voltage (kV)	21–24
Tip-to-collector distance (mm)	60.0
Solution flow rate (mL/h)	0.1, 0.3, 0.6 and 0.8
Scan speed of collector (mm/s)	25.0

**Table 2:** Experimental conditions for fabricating parallel-line patterns at various scan speeds.

Parameter (Units)	Value
Polymer-solvent (concentration, wt %)	PCL-chloroform (8.8 wt %)
Temperature (°C)	20–21
Relative humidity (%)	56–58
Voltage (kV)	21
Tip-to-collector distance (mm)	60.0
Solution flow rate (mL/h)	0.6
Scan speed of collector (mm/s)	10–450

**Table 3:** Experimental conditions for fabricating microfibrous mats at different levels of relative humidity.

Parameter (Units)	Value
Polymer-solvent (concentration, wt %)	PCL-chloroform (8.8 wt %)
Temperature (°C)	20–21
Voltage (kV)	21
Tip-to-collector distance (mm)	60.0
Solution flow rate (mL/h)	0.6
Scan speed of collector (mm/s)	20
Relative humidity (%)	51 and 60
